# AMPK-mediated up-regulation of mTORC2 and MCL-1 compromises the anti-cancer effects of aspirin

**DOI:** 10.18632/oncotarget.7648

**Published:** 2016-02-23

**Authors:** Mei Gao, Qingbin Kong, Hui Hua, Yancun Yin, Jiao Wang, Ting Luo, Yangfu Jiang

**Affiliations:** ^1^ State Key Laboratory of Biotherapy, Section of Oncogene, West China Hospital, Sichuan University, Chengdu, China; ^2^ Laboratory of Stem Cell Biology, West China Hospital, Sichuan University, Chengdu, China; ^3^ Medicine and Pharmacy Research Center, Binzhou Medical University, Yantai, China; ^4^ School of Basic Medicine, Chengdu University of Traditional Chinese Medicine, Chengdu, China; ^5^ Cancer Center, West China Hospital, Chengdu, China

**Keywords:** aspirin, drug resistance, experimental cancer therapy, AMP-activated protein kinase, MCL-1

## Abstract

AMP-activated protein kinase (AMPK) is an important energy sensor that may inhibit cell proliferation or promote cell survival during stresses. Besides cyclooxygenase, AMPK is another target of the nonsteroid anti-inflammatory agent aspirin. Preclinical and clinical investigations demonstrate that aspirin can inhibit several types of cancer such as colorectal adenomas and hepatocellular carcinoma (HCC). However, little is known about the cellular response to aspirin that may lead to aspirin resistance. Here, we show that aspirin induces the expression of MCL-1 in HepG2 and SW480 cells through AMPK-mTOR-Akt/ERK axis. Treatment of HepG2 and SW480 cells with aspirin leads to increased MCL-1 expression, Akt and ERK1/2 phosphorylation. Inhibition of Akt/MEK abrogates the induction of MCL-1 by aspirin. Aspirin activates AMPK, which in turn up-regulates mTORC2 activity, Akt, ERK1/2 phosphorylation and MCL-1 expression. MCL-1 knockdown sensitizes cancer cells to aspirin-induced apoptosis. Combination of aspirin and AMPK, Akt or MEK inhibitor results in more significant inhibition of cell proliferation and induction of apoptosis than single agent. Moreover, sorafenib blocks aspirin-induced MCL-1 up-regulation. Combination of aspirin and sorafenib leads to much more cell death and less cell proliferation than each drug alone. Treatment of HCC and colon cancer xenografts with both aspirin and sorafenib results in more significant tumor suppression than single agent. These data demonstrate that AMPK-mediated up-regulation of mTORC2 and MCL-1 may compromise the anticancer effects of aspirin. Combination of aspirin and sorafenib may be an effective regimen to treat HCC and colon cancer.

## INTRODUCTION

Aspirin is widely prescribed as a painkiller, antipyretic or antiplatelet agent for more than 100 years. The therapeutic effects of aspirin are mediated by its metabolite salicyclic acid. Long-term follow-up study in several trials demonstrates that the nonsteroid anti-inflammatory agent aspirin prevents colorectal adenomas, hepatocellular carcinoma (HCC), melanoma, breast and prostate cancers, and reduces cancer mortality, indicating a promising role of aspirin for cancer prevention [1–3]. In addition to epidemiological studies, preclinical data demonstrate that aspirin may inhibit several types of cancer [4, 5]. However, one study indicates that regular use of aspirin after diagnosis only has beneficial effects among patients with mutated-PIK3CA (phosphatidylinositol-4, 5-bisphosphonate 3-kinase, catalytic subunit alpha) colorectal cancer, but not among patients with wild-type PIK3CA cancer [6]. One mechanism underlying the prevention and inhibition of cancer may be the inhibition of cyclooxygenase (COX) enzymes and reduced production of prostaglandins and other inflammatory mediators [7].

Besides COX, salicyclic acid may target other proteins. Recent studies demonstrate that aspirin may activate AMP-activated protein kinase (AMPK), an important energy sensor in cells [8, 9]. Upon metabolic stress and energy imbalance that lower the intracellular levels of ATP, AMPK is activated to regulate many downstream targets. Activation of AMPK may shape cellular signaling pathways that are involved in tumorigenesis, such as the mTOR and p53 pathways, and inhibit the proliferation of tumor cells [10, 11]. However, AMPK may also reprogram many cellular metabolic processes to restore energy homeostasis thereby promoting cell survival during metabolic stress. Recent study demonstrates that a subunit of AMPK is one of only two genes for which decreased expression resulted in reduced survival of prostate cancer cells [12]. Another study demonstrates that AMPK promotes the survival of cells detached from extracellular matrix and glucose-deprived cells by regulating cellular levels of NADPH, an essential coenzyme for many biosynthetic reactions [13]. In addition, AMPK activation seems to have both anti-tumor and pro-tumor effects in melanoma cells expressing the oncogene BRAF [14].

Although aspirin can inhibit cancer cells, it is unclear whether cancer cells may elicit adaptive response that antagonizes the therapeutic effect of aspirin and leads to drug resistance. Here, we demonstrate that aspirin induces the expression of MCL-1 through AMPK-mTORC2-Akt/ERK axis. Knockdown of AMPK or MCL-1 enhances aspirin-induced apoptosis in HCC and colon cancer cells. The multikinase inhibitor sorafenib blocks aspirin-induced MCL-1 upregulation. Combination of aspirin and sorafenib leads to much more cell death and less cell proliferation rate than each agent alone. Treatment of HCC and colon cancer xenografts with both aspirin and sorafenib results in more significant tumor suppression than single agent. This study reveals a mechanism underlying the promotion of tumor cell survival by AMPK and aspirin resistance.

## RESULTS

### Aspirin induces MCL-1 expression in HepG2 and SW480 cells through Akt/ERK1/2

**Figure 1:**
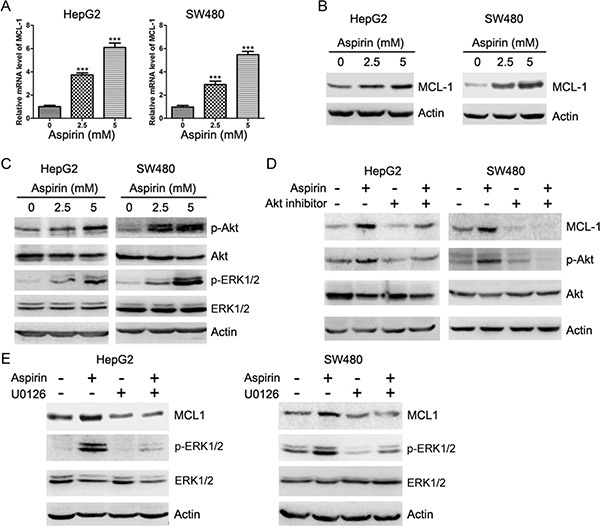
Aspirin induces Akt and ERK1/2 phosphorylation and up-regulates MCL-1 expression (**A**) Quantitative RT-PCR analysis of the effects of aspirin on MCL-1 transcription. The relative levels of MCL-1 mRNA were plotted. *p < 0.001. *n* = 3 in each group. (**B**) Western blot analysis of the effects of aspirin on MCL-1 expression. (**C**) Western blot analysis of the effects of aspirin on Akt and ERK1/2 phosphorylation. (**D**) Western blot analysis of the effects of Akt inhibitor (AKTi, 20 μM) on the induction of MCL-1 expression by 5 mM aspirin. (**E**) Western blot analysis of the effects of MEK inhibitor U0126 (10 μM) on the induction of MCL-1 expression by 5 mM aspirin. A representative of three experiments was shown.

In a preliminary gene expression analysis, we found that MCL-1 transcription was significantly up-regulated by aspirin in breast cancer cell line MDA-MB-231. To determine the effect of aspirin on MCL-1 expression in HCC cells, we treated HepG2 with aspirin, followed by real-time PCR analysis of MCL-1 transcription. Treatment of HepG2 cells with aspirin significantly induced MCL-1 transcription (Figure 1A). Similar effect was detected in colon cancer cells SW480 (Figure 1A). In addition, aspirin induced MCL-1 protein expression in both HepG2 and SW480 cells (Figure 1B). Previous study demonstrates that aspirin has beneficial effect on colorectal cancer patients specifically harboring a mutated PIK3CA [6]. The colon cancer cell lines RKO and HCT-116 harbor mutated PIK3CA. We then detected the effect of aspirin on MCL-1 in RKO and HCT-116 cells. Aspirin inhibited MCL-1 expression in both RKO and HCT-116 cells (Supplementary Figure 1), suggesting that the effects of aspirin on MCL-1 expression vary among different cell lines.

The expression of MCL-1 can be up-regulated by Akt and ERK1/2 [15, 16]. We then detected whether aspirin induced Akt and ERK1/2 phosphorylation in HepG2 and SW480 cells. Aspirin induced Akt and ERK1/2 phosphorylation in both HepG2 and SW480 cells (Figure 1C). Treatment of HepG2 and SW480 cells with Akt inhibitor abrogated the induction of MCL-1 expression by aspirin (Figure 1D). In addition, treatment of HepG2 and SW480 cells with MEK inhibitor blunted the induction of MCL-1 expression by aspirin (Figure 1E). These data suggest that both Akt and ERK1/2 are required for aspirin-induced MCL-1 expression.

### Aspirin stimulates AMPK-Akt/ERK1/2-MCL-1 axis in HepG2 and SW480 cells

**Figure 2:**
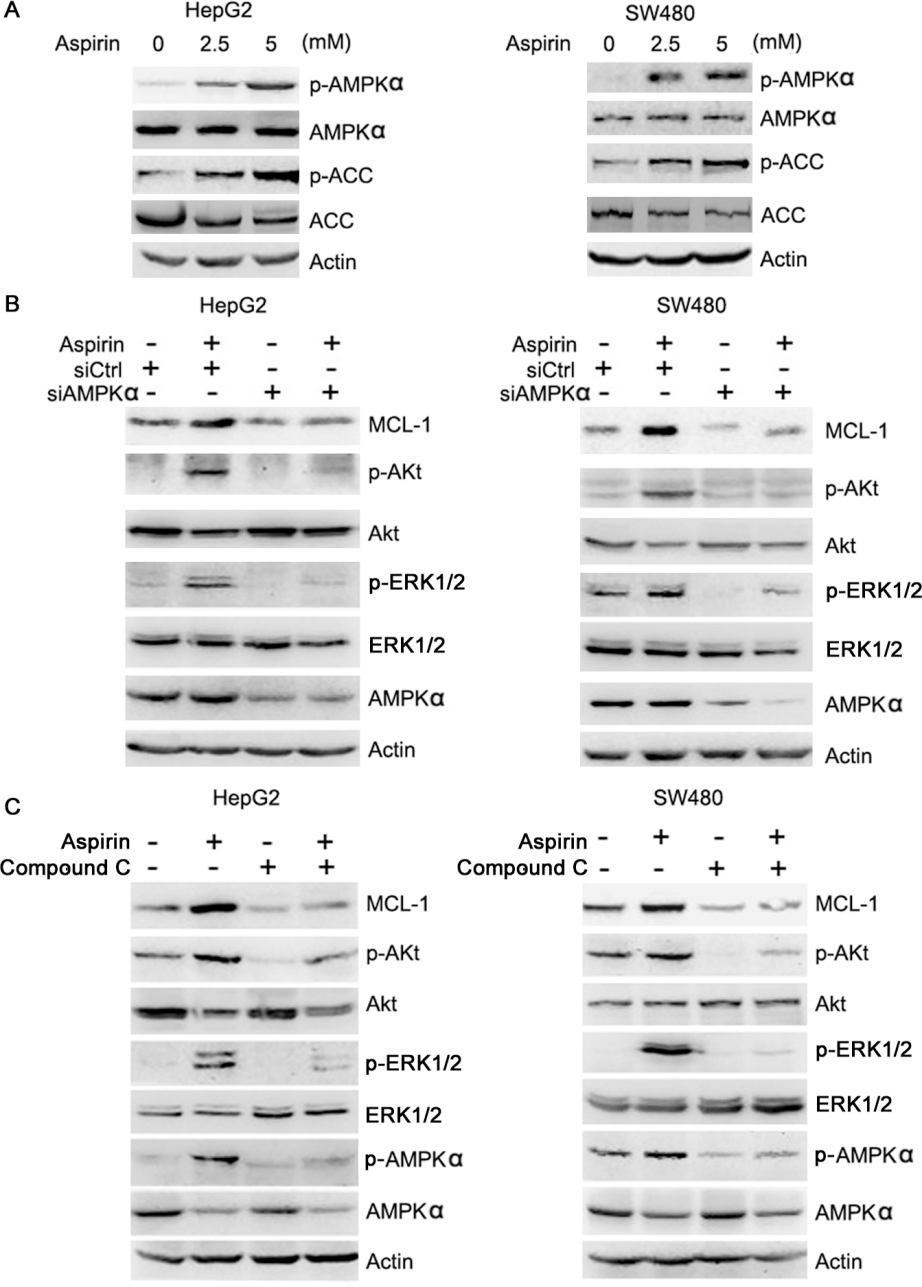
Aspirin activates AMPK, leading to Akt and ERK1/2 phosphorylation (**A**) Western blot analysis of the effects of aspirin on AMPK and ACC phosphorylation. (**B**) Western blot analysis of the effects of AMPK knockdown on the induction of Akt, ERK1/2 phosphorylation and MCL-1 expression by 5 mM aspirin. (**C**) Western blot analysis of the effects of compound C (10 μM) on the induction of Akt, ERK1/2 phosphorylation and MCL-1 expression by 5 mM aspirin. A representative of two independent experiments was shown.

Aspirin is usually known as a COX inhibitor. To determine whether COX inhibition might induce MCL-1 expression, we treated HepG2 and SW480 cells with the COX inhibitor celecoxib, followed by western blot analysis of MCL-1 levels. Treatment with celecoxib did not affect MCL-1 expression in both HepG2 and SW480 cells, suggesting that aspirin may not up-regulate MCL-1 expression through inhibition of COX (Supplementary Figure 2). In addition to inhibition of COX, salicyclic acid can directly or indirectly activate AMPK [8, 9]. To determine whether aspirin induced AMPK activation in HepG2 and SW480 cells, we treated these cells with or without aspirin, followed by western blot analysis of the phosphorylation of AMPK and its target, acetyl-CoA carboxylase (ACC). Indeed, aspirin induced AMPK and ACC phosphorylation in both HepG2 and SW480 cells (Figure 2A). To determine whether AMPK induces MCL-1 expression, we treated HepG2 and SW480 cells with or without 5-Aminoimidazole-4-carboxamide1-β-D-ribofuranoside (AICAR), an AMPK agonist, followed by detection of MCL-1 expression, AMPK, Akt and ERK1/2 phosphorylation. Treatment of HepG2 and SW480 cells with AICAR led to increased AMPK, Akt, ERK1/2 phosphorylation and MCL-1 expression (Supplementary Figure 3). In addition, AMPKα knockdown abrogated the induction of Akt, ERK1/2 phosphorylation and MCL-1 expression by aspirin in both HepG2 and SW480 cells (Figure 2B). Moreover, treatment of HepG2 and SW480 cells with compound C, an AMPK inhibitor, abrogated the induction of AMPK, Akt, ERK1/2 phosphorylation and MCL-1 expression by aspirin (Figure 2C). Collectively, these data demonstrate that aspirin induces MCL-1 expression through AMPK-Akt/ERK axis.

### Aspirin up-regulates Akt and MCL-1 through increasing mTORC2 activity

**Figure 3:**
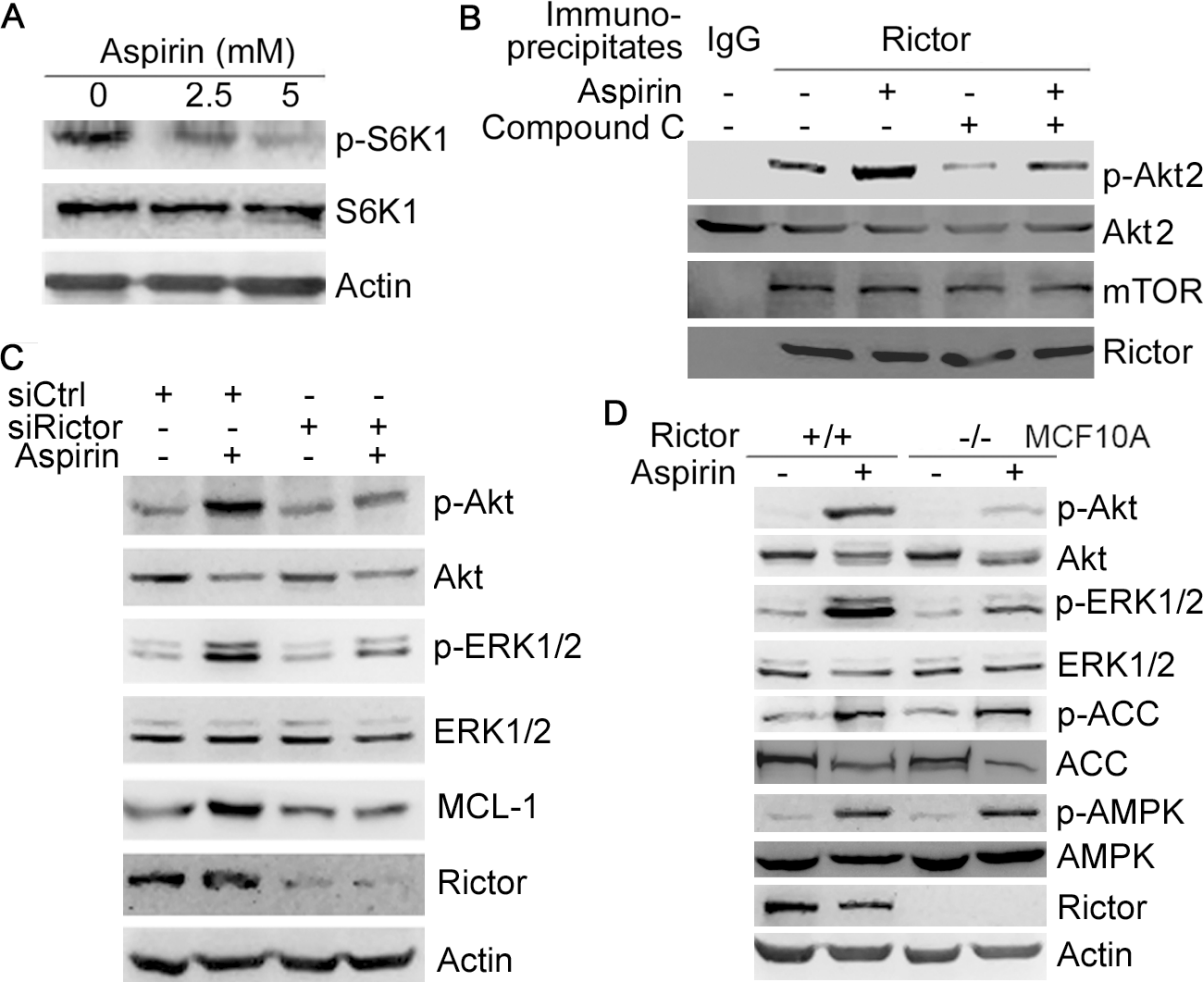
Aspirin induces Akt/ERK phosphorylation and MCL-1 expression by up-regulating mTORC2 activity (**A**) Western blot analysis of the effect of aspirin on S6K1 phosphorylation. (**B**) HepG2 cells were treated with or without 5 mM aspirin and 10 μM compound C, followed by immunoprecipitation of the rictor-mTOR complex. The immunopreciptates were subjected to *in vitro* kinase assay using Akt2 as the substrate, followed by western blot analysis of rictor, mTOR, Akt2 and p-Akt2 (S474). (**C**) Western blot analysis of the effects of rictor knockdown on the induction of Akt, ERK1/2 phosphorylation and MCL-1 expression by 5 mM aspirin. (**D**) Western blot analysis of the induction of Akt and ERK1/2 phosphorylation by 5 mM aspirin in MCF-10A and rictor-null MCF-10A cells. A representative of two or more independent experiments was shown.

Previous studies demonstrate that salicyclic acid can activate AMPK and inhibit mTORC1 [9]. As an effector downstream of mTORC1, S6K1 may inhibit mTORC2 through phosphorylation of rictor and SIN1, two core components of mTORC2 [17, 18]. Thus, inhibition of S6K1 may lead to the paradoxical activation of mTORC2, which promotes Akt phosphorylation. Indeed, treatment of HepG2 cells with aspirin led to decreased S6K1 phosphorylation (Figure 3A). Also, treatment with aspirin resulted in increased mTORC2 activity toward Akt, as determine by *in vitro* kinase assay of rictor immunoprecipitates and western blot analysis of the phosphorylation of mTORC2 target, Akt2 (S474) (Figure 3B). The AMPK inhibitor compound C abrogated the up-regulation of mTORC2 activity by aspirin (Figure 3B). Rictor knockdown resulted in a decrease in aspirin-induced Akt, ERK1/2 phosphorylation and MCL-1 expression (Figure 3C). Whereas aspirin induced Akt and ERK1/2 phosphorylation in MCF-10A cells, it poorly induced Akt and ERK1/2 phosphorylation in rictor-null MCF-10A cells (Figure 3D). Collectively, these data demonstrate that aspirin up-regulates Akt and MCL-1 through AMPK- and mTORC2-dependent mechanism.

### Inhibition of AMPK, Akt, MEK and MCL-1 potentiate aspirin-induced apoptosis

**Figure 4:**
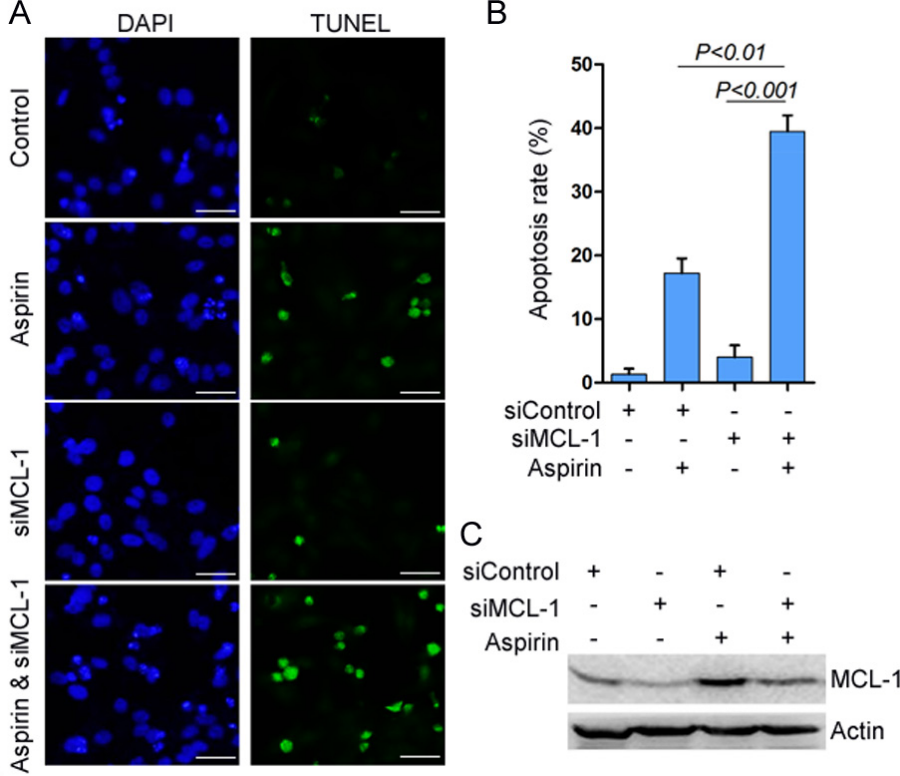
MCL-1 knockdown potentiates aspirin-induced apoptosis (**A**) HepG2 cells were transfected with siControl or siMCL-1, followed by treatment with or without 5 mM aspirin. The cells were subjected to TUNEL assay for detecting apoptosis. *Bar*, 50 μm. (**B**) The apoptosis rate was plotted. The apoptosis rate was plotted. *n* = 3 in each group. (**C**) Western blot analysis of MCL-1 expression. A representative of three independent experiments was shown.

**Figure 5:**
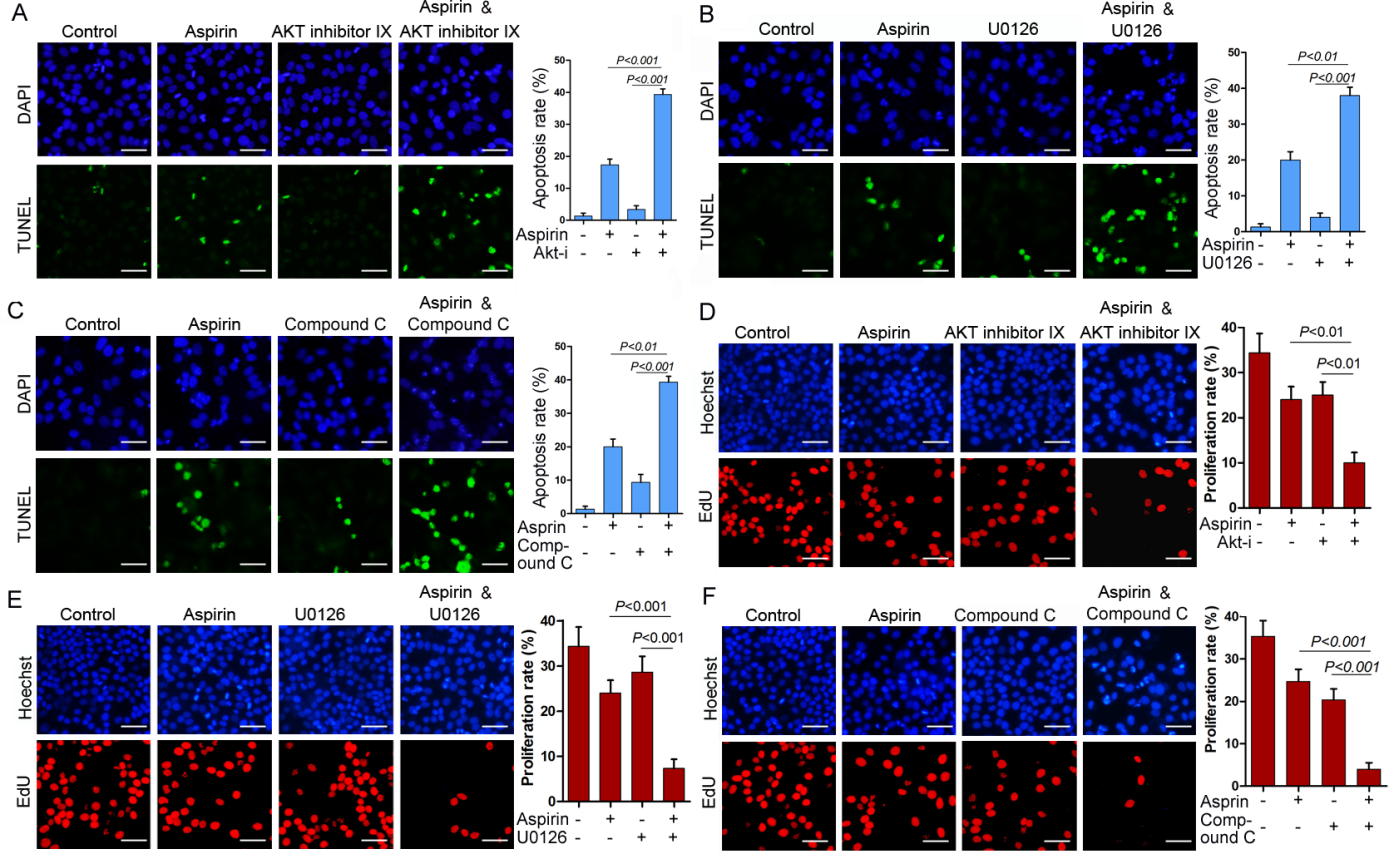
Combination of aspirin and inhibitor of Akt, MEK or AMPK synergistically inhibits cell proliferation and induces apoptosis (**A**) HepG2 cells were treated with or without 5 mM aspirin and 20 μM Akt inhibitor IX, followed by TUNEL analysis of apoptosis. (**B**)HepG2 cells were treated with or without 5 mM aspirin and 10 μM MEK inhibitor U0126, followed by TUNEL analysis of apoptosis. (**C**) HepG2 cells were treated with or without 5 mM aspirin and 10 μM compound C, followed by TUNEL analysis of apoptosis. *Bar*, 50 μm. (**D**) HepG2 cells were treated with or without 5 mM aspirin and 20 μM Akt inhibitor IX, followed by EdU labeling to detect cell proliferation. (**E**) HepG2 cells were treated with or without 5 mM aspirin and 10 μM MEK inhibitor U0126, followed by EdU labeling to detect cell proliferation. (**F**) HepG2 cells were treated with or without 5 mM aspirin and 10 μM compound C, followed by EdU labeling to detect cell proliferation. *Bar*, 50 μm. A representative of two independent experiments was shown.

Given that MCL-1 is an anti-apoptotic protein, we investigated whether MCL-1 knockdown could affect aspirin-induced apoptosis. Indeed, Both TUNEL assay and Hoechst 33342 staining consistently demonstrated that MCL-1 knockdown significantly enhanced aspirin-induced HepG2 cells apoptosis (Figure 4, Supplementary Figure 4). Consistent with above data demonstrating that AMPK, Akt and ERK mediated the induction of MCL-1 by aspirin, combination of aspirin and inhibitor of Akt, MEK or AMPK synergistically inhibited HepG2 and SW480 cells growth (Supplementary Figure 5). To determine whether combination of aspirin and inhibitor of Akt, MEK or AMPK synergistically induced HepG2 cells apoptosis, we detected apoptosis by Hoechst 33342 staining and TUNEL assay. Both Hoechst 33342 staining and TUNEL assay consistently demonstrated that combination of aspirin and inhibitor of Akt, MEK or AMPK synergistically induced HepG2 cells apoptosis (Figure 5A–5C, Supplementary Figure 6). Moreover, combination of aspirin and inhibitor of Akt, MEK or AMPK synergistically induced the cleavage of an effector caspase, caspase-7 in HepG2 cells (Supplementary Figure 6). Meanwhile, combination of aspirin and inhibitor of Akt, MEK or AMPK synergistically induced the cleavage of poly (ADP-ribose) polymerase 1 (PARP1), a target of the effector caspase (Supplementary Figure 6). Combination of aspirin and inhibitor of Akt, MEK or AMPK also synergistically induced caspase-7 cleavage and apoptosis in SW480 cells (Supplementary Figure 7). In addition, combination of aspirin and inhibitor of Akt, MEK or AMPK resulted in much lesser cell proliferation than single agent in both HepG2 and SW480 cells (Figure 5D–5F, and Supplementary Figure 8). Collectively, these data suggest that activation of AMPK, Akt and ERK may help the proliferation and survival of aspirin-treated cancer cells. While MCL-1 knockdown sensitizes cells to aspirin-induced apoptosis, it did not affect proliferation (data not shown), suggesting that MCL-1 conferred resistance to apoptosis downstream of AMPK, Akt and ERK.

### Combination of aspirin and sorafenib synergistically inhibits cell growth

**Figure 6:**
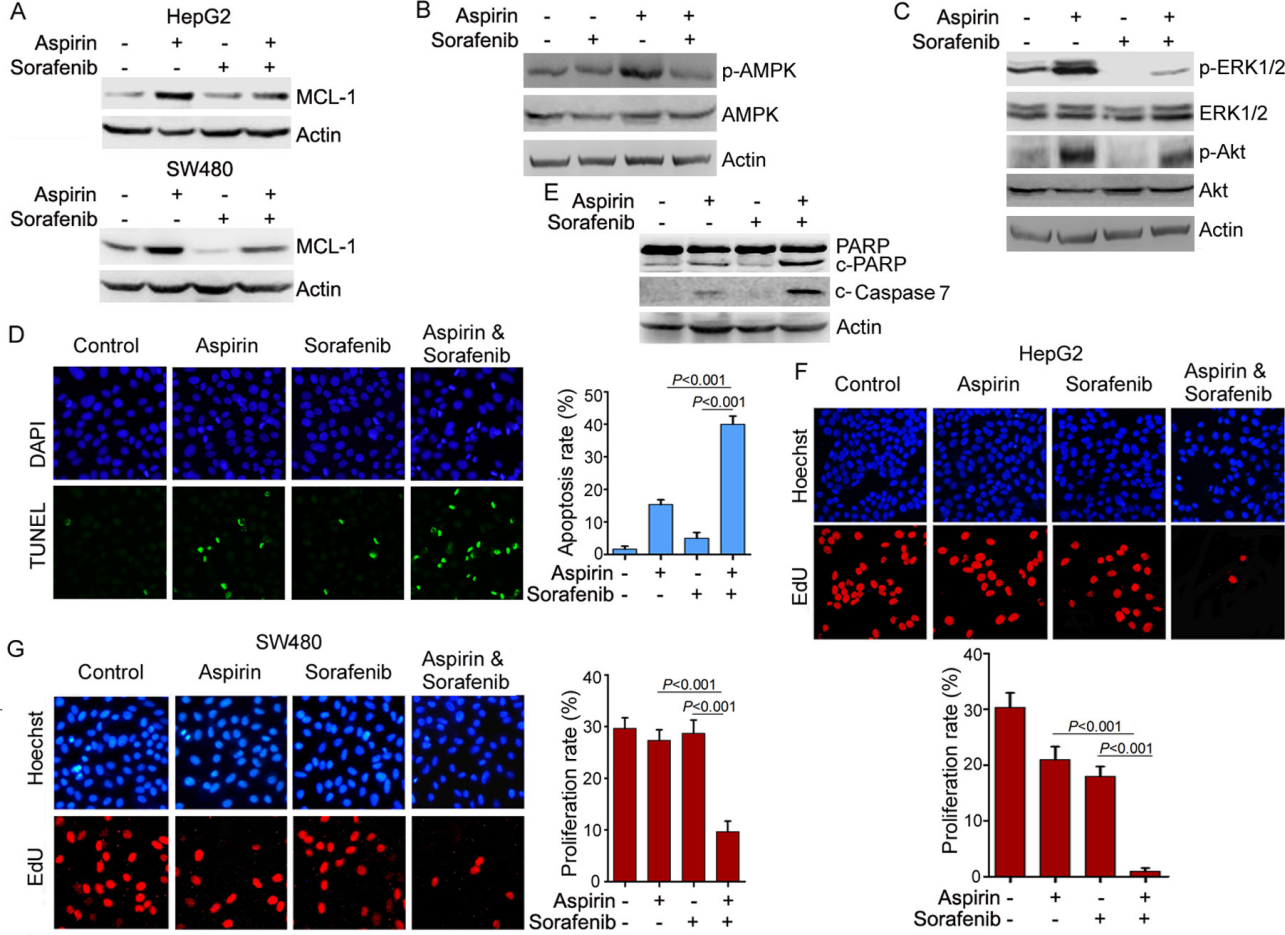
Sorafenib inhibits the induction of MCL-1 expression by aspirin and potentiates the anti-cancer activity of aspirin (**A**) Western blot analysis of the effects of sorafenib (5 μM) and aspirin (5 mM) on MCL-1 expression. (**B**) Western blot analysis of the effects of sorafenib (5 μM) and aspirin (5 mM) on AMPKα phosphorylation in SW480 cells. (**C**) Western blot analysis of the effects of sorafenib (5 μM) and aspirin (5 mM) on Akt and ERK1/2 phosphorylation in SW480 cells. (**D**) TUNEL analysis of the effects of sorafenib and aspirin on apoptosis. The apoptosis rate was plotted. (**E**) Western blot analysis of the effects of sorafenib (5 μM) and aspirin (5 mM) on caspase-7 and PARP cleavage. (**F**) EdU-labeling analysis of the effects of sorafenib (5 μM) and aspirin (5 mM) on HepG2 cell proliferation. The proliferation rate was plotted. (**G**) EdU-labeling analysis of the effects of sorafenib (5 μM) and aspirin (5 mM) on SW480 cell proliferation. The proliferation rate was plotted. *n* = 3 in each group. A representative of two independent experiments was shown.

Previous studies demonstrated that the multikinase inhibitor sorafenib could inhibit MCL-1 expression [19]. To determine whether sorafenib affected aspirin-induced MCL-1 expression, HepG2 and SW480 cells were treated with or without aspirin and sorafenib, followed by western blot analysis of MCL-1 expression. Sorafenib suppressed the induction of MCL-1 expression by aspirin in both HepG2 and SW480 cells (Figure 6A). Also, the induction of AMPK, Akt and ERK1/2 phosphorylation by aspirin was inhibited by sorafenib (Figure 6B, 6C). Moreover, combination of aspirin and sorafenib synergistically inhibited HepG2 and SW480 cells growth (Supplementary Figure 9). Next, we investigated the effect of sorafenib on aspirin-induced apoptosis. While sorafenib alone poorly induced HepG2 cells apoptosis, it significantly enhanced aspirin-induced apoptosis (Figure 6D and Supplementary Figure 10). Combination of aspirin and sorafenib led to more caspase-7 and PARP1 cleavage than single agent (Figure 6E). Caspase inhibitor blunted the stimulation of aspirin-induced apoptosis by sorafenib, whereas it did not block apoptosis induced by aspirin alone in HepG2 cells (Supplementary Figure 10). Similar effect was detected in SW480 cells (Supplementary Figure 11). These data indicate that sorafenib stimulates aspirin-induced apoptosis via enhancing caspase activation, while aspirin alone induces apoptosis through caspase-independent mechanism.

In addition, we investigated the effect of combination of aspirin and sorafenib on cell proliferation. While treatment of HepG2 and SW480 cells with aspirin or sorafenib alone had little effect on cell proliferation, combination of aspirin and sorafenib significantly inhibited cell proliferation (Figure 6F, 6G).

### Combination of aspirin and sorafenib synergistically inhibits tumor growth

**Figure 7:**
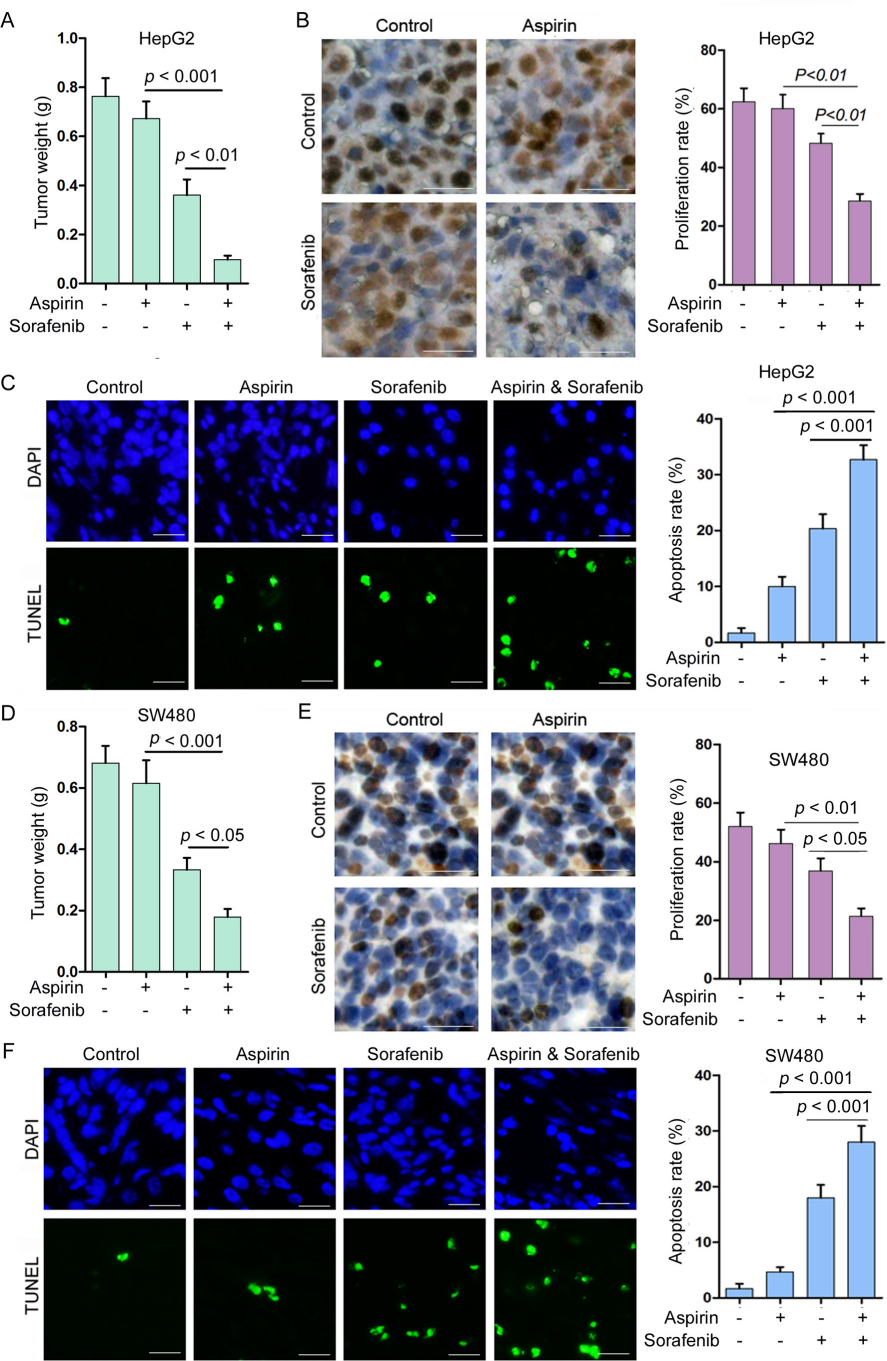
Combination of aspirin and sorafenib inhibits tumor growth (**A**) HepG2 cells were subcutaneously injected into nude mice. The mice were treated with or without aspirin (100 mg/kg/day) and sorafenib (15 mg/kg/day). The effect of aspirin and sorafenib on HepG2 tumor weight was plotted (*n* = 10 for each group). (**B**) Immunohistochemical analysis of Ki-67 positivity in HepG2 tumors treated with or without aspirin and sorafenib. *Bar*, 50 μm. The proliferation rate was plotted. (**C**) TUNEL analysis of apoptosis in HepG2 tumors treated with or without aspirin and sorafenib. *Bar*, 50 μm. The apoptosis rate was plotted. (**D**) SW480 cells were subcutaneously injected into nude mice. The mice were treated with or without aspirin (100 mg/kg/day) and sorafenib (15 mg/kg/day). The effect of aspirin and sorafenib on SW480 tumor weight was plotted (*n* = 10 for each group). (**E**) Immunohistochemical analysis of Ki-67 positivity in SW480 tumors treated with or without aspirin and sorafenib. *Bar*, 50 μm. The proliferation rate was plotted. (**F**) TUNEL analysis of apoptosis in SW480 tumors treated with or without aspirin and sorafenib. *Bar*, 50 μm. The apoptosis rate was plotted. A representative of two independent experiments was shown.

To determine the effect of combination of aspirin and sorafenib on tumor growth, we tested the tumor suppressive effect of aspirin and sorafenib in HepG2 xenografts. Treatment with sorafenib moderately inhibited tumor growth, while treatment with aspirin alone had little effect on tumor growth (Figure 7A, and Supplementary Figure 12A). Combination of aspirin and sorafenib inhibited tumor growth more significantly than sorafenib alone (Figure 7A, and Supplementary Figure 12A). In addition, we detected the levels of MCL-1 in these tumor xenografts. Aspirin up-regulated MCL-1 expression, while treatment with sorafenib suppressed aspirin-induced MCL-1 expression (Supplementary Figure 12B). Combination of aspirin and sorafenib inhibited tumor cells proliferation more significantly than sorafenib alone, while aspirin alone had no effect on cell proliferation (Figure 7B). Moreover, the apoptosis rate in tumors treated with both aspirin and sorafenib was significantly higher than that in tumors treated with aspirin or sorafenib alone (Figure 7C).

In addition, we tested the effects of aspirin and sorafenib on colon cancer. Treatment with sorafenib moderately inhibited SW480 xenografts growth, while treatment with aspirin alone had little effect on tumor growth (Figure 7D, and Supplementary Figure 11C). Combination of aspirin and sorafenib inhibited tumor growth more significantly than sorafenib alone (Figure 7D, and Supplementary Figure 12C). Treatment with sorafenib suppressed aspirin-induced MCL-1 expression (Supplementary Figure 12D). Combination of aspirin and sorafenib inhibited tumor cells proliferation and induced apoptosis more significantly than single agent (Figure 7E, 7F). These data indicate that combination of aspirin and sorafenib may be a better choice for treating cancer than sorafenib alone.

## DISCUSSION

The nonsteroid antiinflammatory agent aspirin is a promising anti-aging or cancer chemoprevention agent [20, 21]. In addition, the favorable outcome that has been associated with treatment of colorectal cancer and hepatocellular carcinoma with aspirin suggests that aspirin may be a promising agent for adjuvant therapy [7, 22, 23]. Besides inhibition of COX, aspirin has a diversity of tumor suppressive effects through COX-independent mechanisms [24, 25]. Despite a lot of epidemiological and preclinical studies that test the cancer preventive role and tumor suppressive role of aspirin, little is known about the roadblocks that counteract the anticancer effect of aspirin.

The current study demonstrates that aspirin could up-regulate MCL-1 expression, which antagonizes cell apoptosis. AMPK-mTOR-Akt/ERK axis is responsible for the induction of MCL-1 by aspirin. AMPK can inhibit mTORC1. Previous studies have demonstrated that inhibition of mTORC1 may paradoxically lead to increased mTORC2 activity through multiple mechanisms [18, 26]. While mTOR is usually known as a serine/threonine kinase, we recently find that mTOR has tyrosine kinase activity and identify IGF-IR and insulin receptor as novel targets of mTORC2 [27]. Therefore, mTOR belongs to dual-specificity kinase that targets serine, threonine and tyrosine [27]. Whereas aspirin inhibits mTORC1, it stimulates mTORC2 kinase activity in AMPK-dependent manner. While AMPK reportedly inhibits cell proliferation [10, 11], it is required for cell survival under the conditions of elevated MYC levels [25]. In addition, decreased expression of a subunit of AMPK reduces survival of prostate-cancer cells, but has no effect on normal prostate cells [12]. The effects of AMPK activation on cell proliferation and survival may be context-dependent. We demonstrate here, in the context of aspirin treatment, activation of AMPK may positively regulate cancer cell proliferation and survival through Akt, ERK and MCL-1. Up-regulation of MCL-1 represents another mechanism underlying the promotion of cell survival by AMPK. Aspirin-treated tumor cells appear to be addictive to AMPK-Akt/ERK activation, since inhibition of AMPK, Akt or ERK greatly inhibits cell proliferation and promotes apoptosis.

While aspirin reportedly inhibits MCL-1 expression in leukemia cells and oral squamous carcinoma cells [29, 30], the current study demonstrates that aspirin up-regulates MCL-1 expression in colon cancer cell line SW480, but down-regulates MCL-1 in aspirin sensitive RKO and HCT-116 cells. Although a report suggests that aspirin down-regulates MCL-1 in HepG2 cell line [31], the current study demonstrates that aspirin up-regulates MCL-1 in HepG2 cells. In addition, a study in some colon cancer cells found that no change in MCL-1 expression after treatment of these cells with aspirin [32]. Hence, aspirin may regulate MCL-1 in context- or cell type-dependent manner. Inhibition of COX2, one of the targets of aspirin, reportedly inhibits PI3K/Akt kinase activity in epithelial ovarian cancer [33]. Consistent with the current study, Feng and collegues reported that aspirin could induce Akt and ERK1/2 phosphorylation in HepG2 cells [34]. Our current study demonstrates that activation of AMPK contributes to the induction of Akt phosphorylation by aspirin. Therefore, the extent to which COX2 is inhibited and AMPK is activated may dictate the fate of cancer cells exposed to aspirin. Given that the metabolite of aspirin, rather than aspirin itself directly activates AMPK, it is possible that the metabolism of aspirin in different cell types may affect its biological outcome. Recent studies suggest that the genetic backgrounds of tumors or individuals may affect the response to aspirin, either in chemoprevention or chemotherapy setting [6, 35]. Change in Akt, ERK1/2 phosphorylation and MCL-1 levels may represent additional predictive biomarkers for the responsiveness to aspirin.

Drug resistance is a critical problem in cancer therapy. The same may be true for cancer prevention, especially after long-term administration of the drugs. The current study demonstrates that combination of aspirin and sorafenib has synergistical effect on HCC and colon cancer. While aspirin may paradoxically promote Akt/ERK activation and MCL-1 expression, sorafenib can antagonize the unwanted effects of aspirin. Combination of aspirin and sorafenib reportedly suppresses metastasis of HCC [36]. Currently, sorafenib is the only molecular-targeted drug to treat HCC patients. It remains to know whether combination of aspirin and sorafenib has beneficial effects in clinical setting.

## MATERIALS AND METHODS

### Reagents

The biochemical reagents used were as follows: aspirin (Sigma-Aldrich, St. Louis, MO); sorafenib, celecoxib (LC lab, Woburn, MA); AKT inhibitor IX, Compound C (Merk Millipore Corporation, Darmstadt, Germany), AICAR, U0126 (Beyotime, Jiangsu, China); Torin2 (Tocris Bioscience, Bristol, UK); recombinant Akt2 protein (BPS Bioscience, San Diego, CA). The antibodies used were as follows: anti-MCL-1, Akt, p-Akt (S473), ERK1/2, p-ERK1/2(T202/Y204), AMPKα, p-AMPKα (T172), p-ACC (S79), p-mTOR (S2481), p-S6K1 (S371), c-Caspase-7, PARP (Cell Signaling Technology, Beverly, MA); rictor (Merk Millipore Corporation, Darmstadt, Germany); mTOR, Ki67 (Burlingame, CA, USA); actin (Proteintech, Chicago, IL, USA).

### Cell culture

Hepatoma cell lines HepG2, colon cancer cell line SW480 were obtained from Cell Lines Bank, Chinese Academy of Science (Shanghai, China). The cells were maintained in DMEM containing 10% fetal bovine serum, 100 U/ml of penicillin and 100 mg/ml streptomycin sulfate and incubated at 37°C in a humidified atmosphere of 5% CO_2_. MCF-10A and rictor-null MCF-10A cells were purchased from Sigma, and culture in DMEM/F12 medium containing 5% horse serum, 2.5 mM L-glutamine, 10 mg/mL human insulin, 0.5 mg/mL hydrocortisone, 10 ng/mL EGF, and 100 ng/mL cholera toxin, 100 U/ml of penicillin and 100 mg/ml streptomycin sulfate.

### Quantitative real-time PCR analysis

Treated cells were lysed in plates after removing of medium. Total RNAs were obtained using Trizol Reagent (Thermo Fisher Scientific, Waltham, MA). cDNAs were prepared with random hexamers from mRNA, using the AMV reverse-transcription kit (Promega Biotechnology, Madison, WI). The cDNA amplification reactions were performed using the PowerSYBR Green PCR master mix (Thermo Fisher Scientific, Waltham, MA). Quantitative PCR reactions were performed on the iQ5^™^ (BIO- RAD, Hercules, CA).

### RNA interference

All siRNAs were custom-synthesized products of Ribobio Co., Ltd. (Guangzhou, China). The sequences of AMPKα1, rictor and MCL-1 siRNA used are 5′-UUACUUCUGGUGCAGCAUAdTdT-3′, 5′-ACUUGUGAAGAAUCGUAUCdTdT-3′, 5′-AAGUAU CACAGACGUUCUCdTdT-3′, respectively. The double-stranded siRNA was dissolved in DEPC-treated water. LipofecTAMINE 2000 reagent (Thermo Fisher Scientific, Waltham, MA) and 50 nM siRNA duplex were separately diluted in 250 μl of OPTI-MEM^®^I Reduced Serum Medium and incubated at room temperature. After 5 minutes, the two solutions were mixed together. The mixture was incubated at room temperature for 20 minutes and then added into cell culture. 48 hours after transfections, cells were harvested for further experiments.

### Western blotting

Cultured cells were washed with phosphate buffered saline and then lysed with ice-cold lysis buffer (1% Triton X-100, 40 mM HEPES pH 7.5, 120 mM NaCl, 1 mM EDTA, 10 mM pyrophosphate, 10 mM glycerophosphate, 50 mM NaF, 0.5 mM orthovanadate) containing protease and phosphatase inhibitors (Roche, Indianapolis, IN). Cell lysates were incubated on ice for 30 minutes and then centrifuged for 20 minutes at 12,000 g. Protein concentrations were determined using the BCA Protein Assay Kit (Thermo Fisher Scientific, Waltham, MA). Protein samples were boiled in 1 × loading buffer for 10 minutes, then separated on polyacrylamide gels and transferred to PVDF membrane (Merk Millipore Corporation, Darmstadt, Germany). Membranes were incubated with primary antibodies overnight at 4°C and appropriate HRP-secondary antibodies for 1 hour at room temperature. Detection was performed with chemiluminescent agents. Images were acquired by Alpha Innotech's FluorChem imaging system.

### Co-immunoprecipitation

Treated cells were lysed in lysis buffer (0.3% CHAPS, 40 mM HEPES pH 7.5, 120 mM NaCl, 1 mM EDTA, 10 mM pyrophosphate, 10 mM glycerophosphate, 50 mM NaF, 0.5 mM orthovanadate) containing protease and phosphatase inhibitors. 3 μg of the appropriate primary antibodies or IgG were added to the protein concentrations and incubated at 4°C with rotation for 24 hours. 40 μl slurry of protein G agarose beads were added into the protein concentrations for 3 hours at 4°C. After washing in the lysis buffer four times, immunoprecipitated proteins were analyzed by western blotting.

### *In vitro* kinase assays

The immunoprecipitated target protein samples were washed once with cold kinase buffer (25 mM Hepes pH 7.5, 100 mM potassium acetate, 1 mM MgCl_2_). Then the recombinant Akt2 protein and 500 μM ATP were added into the immunoprecipitated protein samples and incubated at 37°C. One hour later, the reaction was stopped with the addition of 30 μl loading buffer and boiled for 10 minutes. The phosphorylation of substrate protein was analyzed by Western blotting.

### Cell apoptosis assay

Cell apoptosis was measured by Hoechst 33342 or TUNEL assay (Beyotime, Jiangsu, China). For Hoechst 33342 staining, the treated cells were incubated with Hoechst 33342 solution at 4°C for 5 minutes, followed by detecting under a fluorescence microscope (Leica Microsystems Gmbh, Wetzlar, Germany). Apoptotic cells were stained highlight blue fluorescence, while live cells were stained weak blue fluorescence. Quantification of apoptotic cells was performed by acquiring the images in random fields and counting cells in each well. For TUNEL assay, 4% paraformaldehyde was added in the treated cells for 1 hour. Next, the cells were permeabilized by 0.25% Triton X-100 in PBS for 2 minutes, followed by incubating with TUNEL solution and 4′, 6-diamidino-2-phenylindole (DAPI) solution at 37°C for 60 minutes in dark. The cells were detected by the fluorescence microscope. The nuclei of apoptotic cells were stained highlight green fluorescence, and all cells showed blue fluorescence with DAPI. Quantification of all cells and apoptotic cells in same fields were performed by acquiring the images in random fields and counting cells in four random fields in each well.

### Xenograft tumor growth

The animal care was in accordance with institution guidelines. 5 × 10^6^ cultured cancer cells in 100 μl of PBS were subcutaneously injected into 5-week-old BALB/c nude mice. After about 5 days, when the tumor size reached approximately 2–4 mm, the mice were orally administrated with aspirin (100 mg/kg/day), sorafenib (15 mg/kg/day) or both daily. The control group administrated with vehicle only. The size of the tumor was measured per three days using a vernier caliper. About 30 days after the beginning of treatment, the mice were sacrificed, and the tumors were resected. The tumor tissues were subjected to immunohistochemical analysis and western blotting.

### Immunohistochemistry

Immunohistochemistry (IHC) was performed on tissue sections from formalin-fixed and paraffin-embedded tissue blocks of the xenograft models. The tissue sections were mounted on slides and deparaffinized by xylene followed by rehydration using graded ethanol. Next, the slides were subjected to antigen retrieval and incubated with anti-Ki67 primary antibody overnight at 4°C and biotin-labeled secondary antibody for 15 minutes at room temperature, followed by incubating with streptavidin/horseradish peroxidase (HRP) for 15 minutes, staining with diaminobenzidine chromagen, and counterstaining with hematoxylin. Finally, the slides were detected by the light microscope. The cells expressing Ki67 were stained brown, and all cells showed blue with hematoxylin. Quantification of all cells and the Ki67 positive cells in same fields were performed by acquiring the images in random fields and counting cells in four random fields in each slide.

## SUPPLEMENTARY MATERIALS FIGURES



## Abbreviations

AMPK: AMP-activated protein kinase
mTORC2: mTOR complex 2
