# Supplementary material for: AMPK-mediated up-regulation of mTORC2 and MCL-1 compromises the anti-cancer effects of aspirin
Source: Oncotarget. 2016 Feb 23;7(13):16349–61. doi: 10.18632/oncotarget.7648 (PMC4941319; doi:10.18632/oncotarget.7648)
Supplement: Supplementary file 1 [file oncotarget-07-16349-s001.pdf]

AMPK-mediated up-regulation of mTORC2 and MCL-1 compromises the anti-cancer effects of aspirin

Supplementary Materials

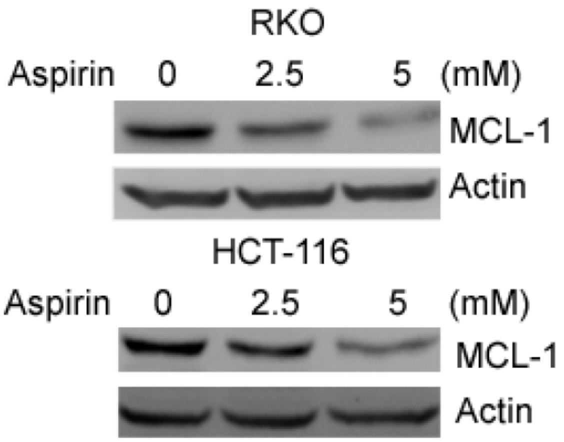

Supplementary Figure S1: Aspirin inhibits MCL-1 expression in RKO and HCT-116 cells. RKO and HCT-116 cells were treated with or without aspirin for 24 h, followed by western blot analysis of MCL-1 expression.

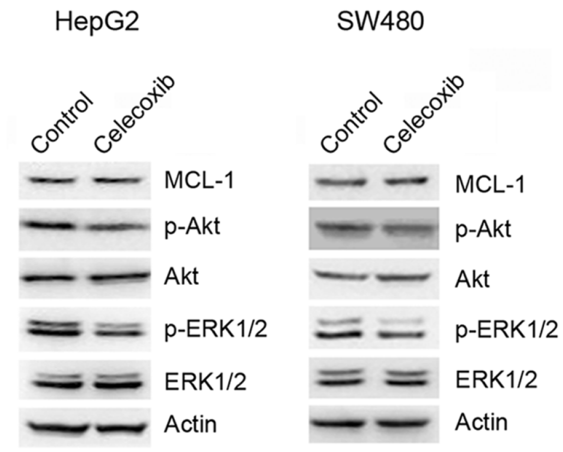

Supplementary Figure S2: The COX inhibitor celecoxib does not affect MCL-1 expression. HepG2 and SW480 cells were treated with or without 20 µM celecoxib for 48 h, followed by western blot analysis of Akt, ERK1/2 phosphorylation and MCL-1 expression.

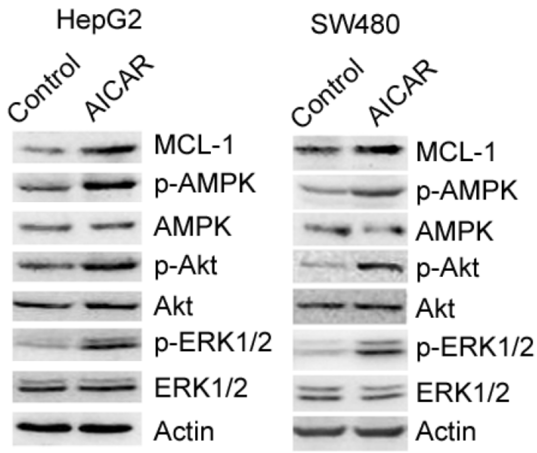

Supplementary Figure S3: The AMPK agonist AICAR induces Akt, ERK1/2 phosphorylation and MCL-1expression. HepG2 and SW480 cells were treated with or without 1 mM AICAR for 48 h, followed by western blot analysis of Akt, ERK1/2 phosphorylation and MCL-1 expression.

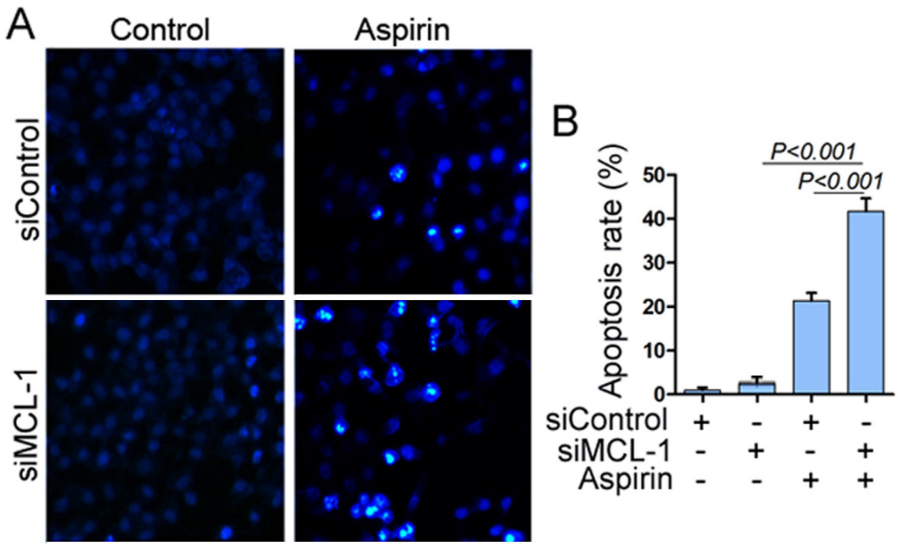

Supplementary Figure S4: MCL-1 knockdown potentiates aspirin-induced apoptosis. (**A**) HepG2 cells were transfected with siControl or siMCL-1, followed by treatment with or without 5 mM aspirin. The cells were subjected to Hoechst 33342 staining for detecting apoptosis. Apoptotic cells exhibited strong fluorescence or condensed nuclei. (**B**) The apoptosis rate was plotted.

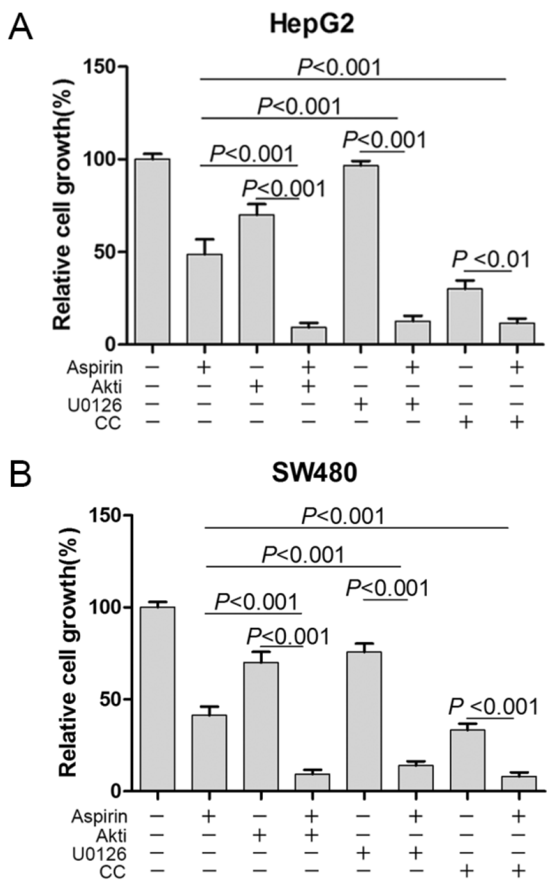

Supplementary Figure S5: Combination of aspirin and Akt inhibitor, MEK inhibitor or AMPK inhibitor inhibits cell growth. HepG2 and SW480 cells were treated with or without 5 mM aspirin and 20 µM Akt inhibitor IX, 10 µM U0126 or 10 µM compound C for 48 h, followed by detection of cell viability with CCK-8 reagents.

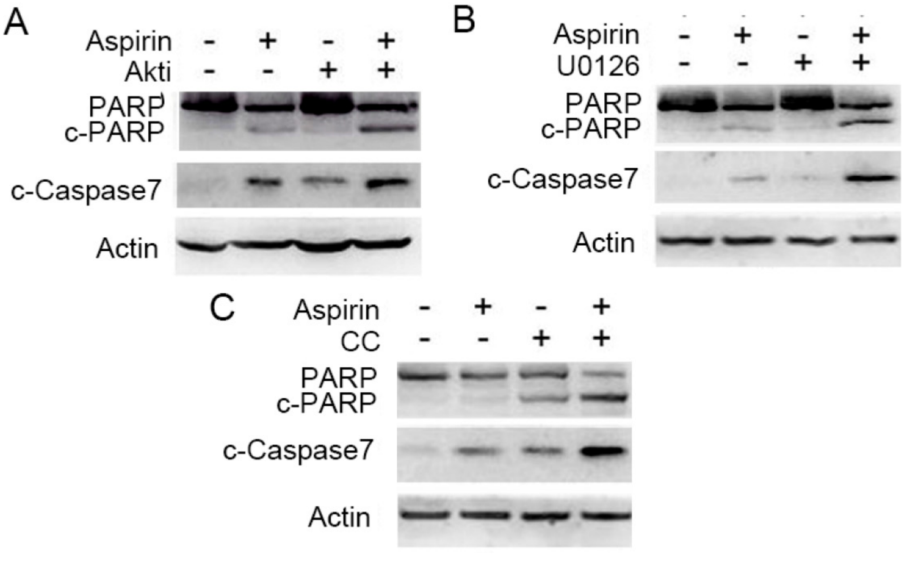

Supplementary Figure S6: Combination of aspirin and Akt inhibitor, MEK inhibitor or AMPK inhibitor induces caspase-7 and PARP cleavage in HepG2 cells. (**A**) HepG2 cells were treated with or without 5 mM aspirin and 20 µM Akt inhibitor IX for 48 h, followed by western blot analysis of caspase-7 and PARP cleavage. (**B**) HepG2 cells were treated with or without 5 mM aspirin and 10 µM U0126 for 48 h, followed by western blot analysis of caspase-7 and PARP cleavage. (**C**) HepG2 cells were treated with or without 5 mM aspirin and 10 µM compound C for 48 h, followed by western blot analysis of caspase-7 and PARP cleavage.

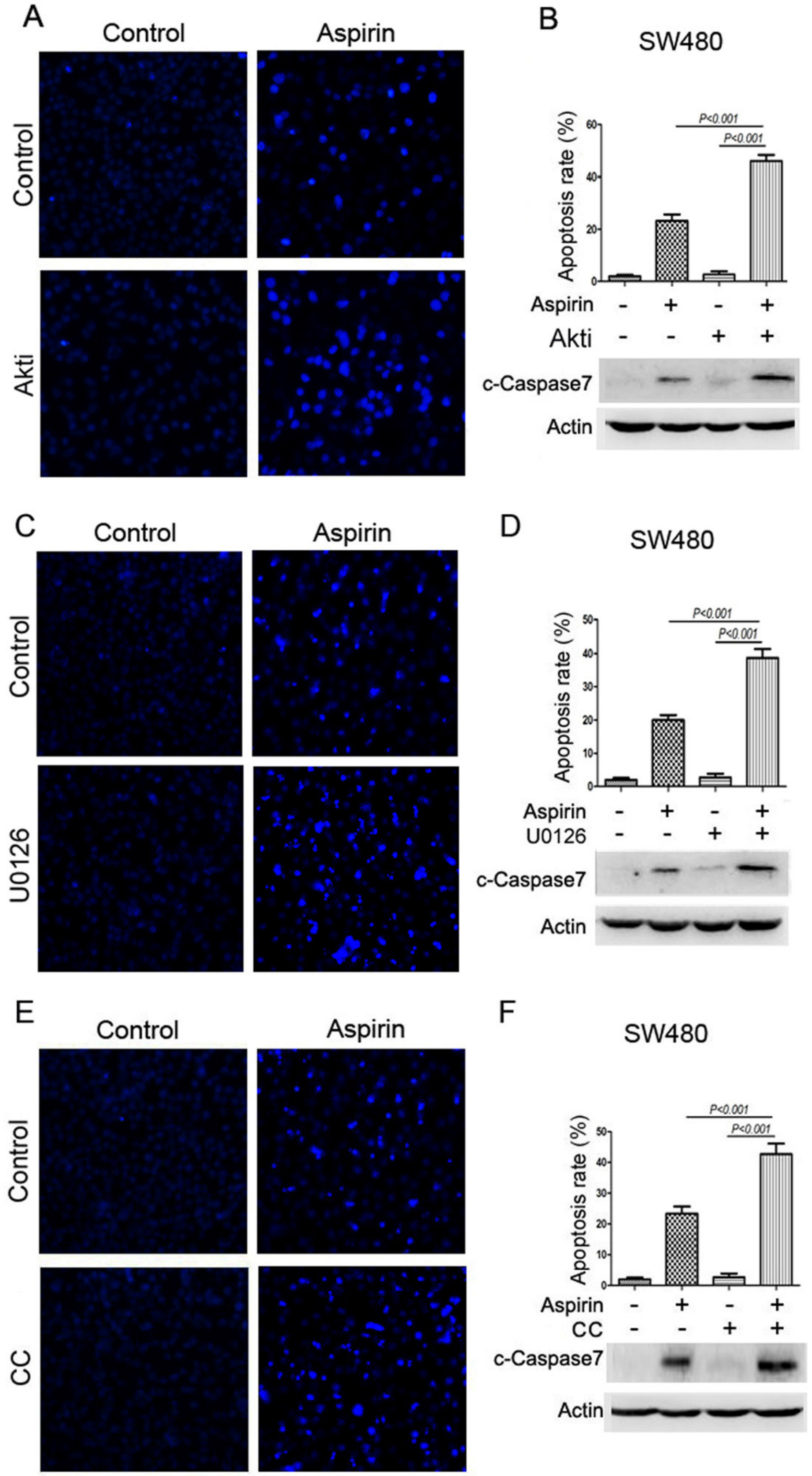

Supplementary Figure S7: Combination of aspirin and Akt inhibitor, MEK inhibitor or AMPK inhibitor induces SW480 cells apoptosis. (**A**) SW480 cells were treated with or without 5 mM aspirin and 20 μM Akt inhibitor IX for 48 h, followed by detection of apoptosis with Hoechst 33342. (**B**) The apoptosis rate in (a) was plotted. The effects on caspase-7 cleavage were also shown. (**C**) SW480 cells were treated with or without 5 mM aspirin and 10 μM U0126 for 48 h, followed by detection of apoptosis with Hoechst 33342. (**D**) The apoptosis rate in (C) was plotted. The effects on caspase-7 cleavage were also shown. (**E**) SW480 cells were treated with or without 5 mM aspirin and 10 μM compound C for 48 h, followed by detection of apoptosis with Hoechst 33342. (**F**) The apoptosis rate in (E) was plotted. The effects on caspase-7 cleavage were also shown.

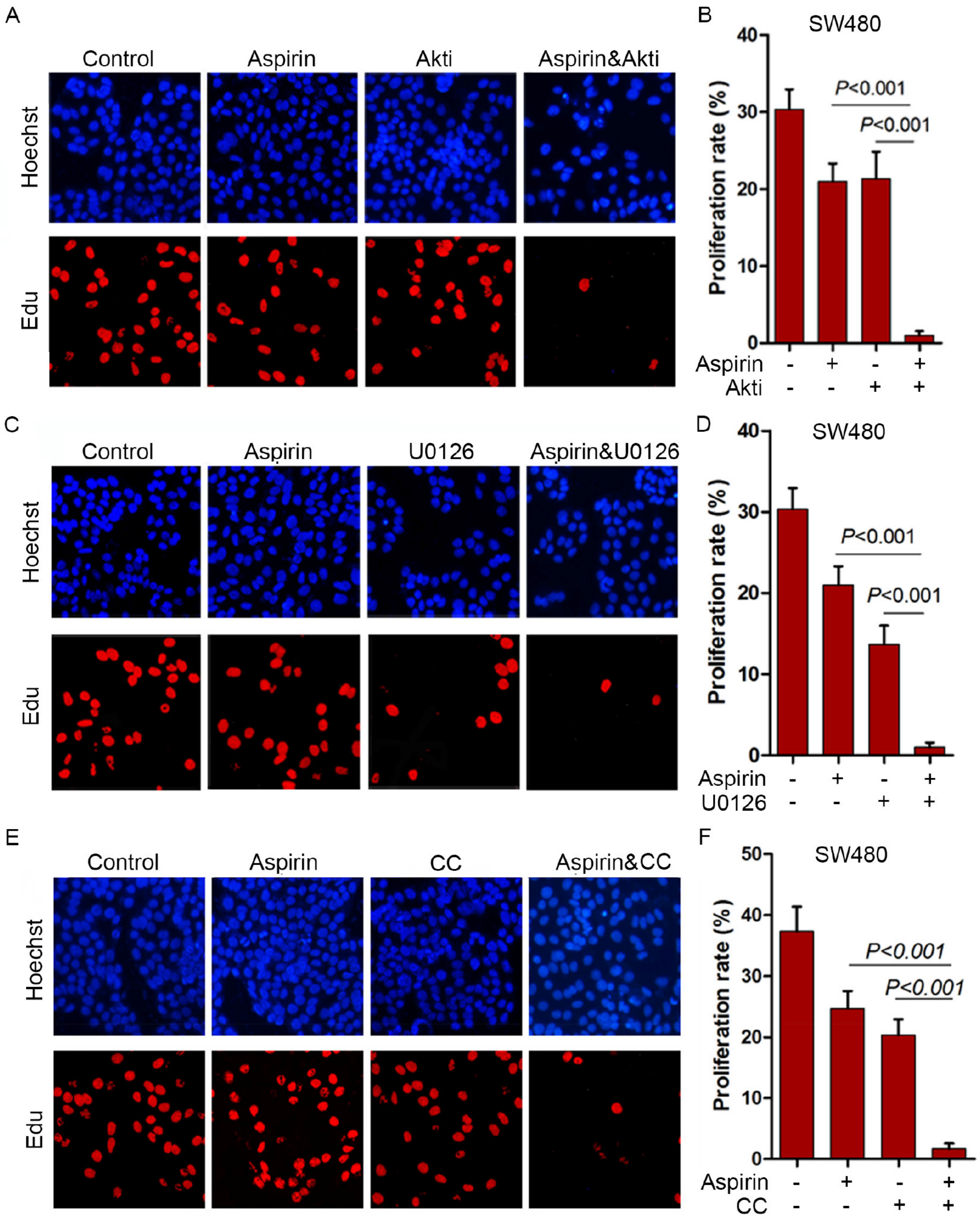

Supplementary Figure S8: Combination of aspirin and Akt inhibitor, MEK inhibitor or AMPK inhibitor inhibits SW480 cells proliferation. (**A**) SW480 cells were treated with or without aspirin and Akt inhibitor IX for 48 h, followed by EdU labeling to detect cell proliferation. (**B**) The proliferation in (A) was plotted. (**C**) SW480 cells were treated with or without 5 mM aspirin and 10 μM U0126 for 48 h, followed by EdU labeling to detect cell proliferation. (**D**) The proliferation rate in (C) was plotted. (**E**) SW480 cells were treated with or without 5 mM aspirin and 10 μM compound C for 48 h, followed by EdU labeling to detect cell proliferation. (**F**) The proliferation rate in (E) was plotted.

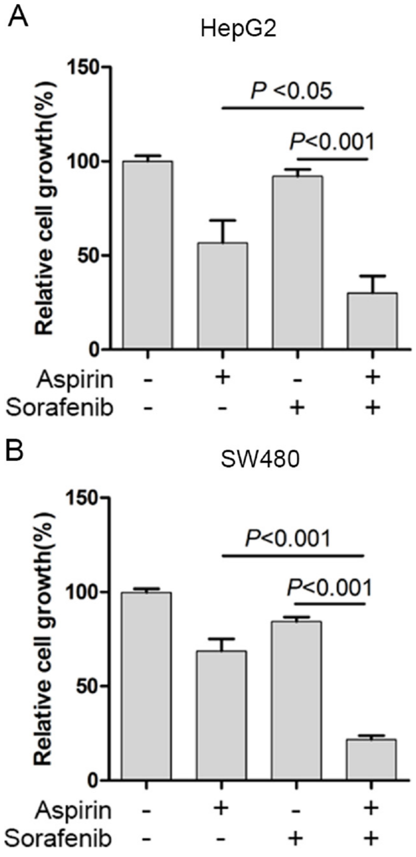

Supplementary Figure S9: Combination of aspirin and sorafenib inhibits cell growth. HepG2 and SW480 cells were treated with or without 5 mM aspirin and 5 μM sorafenib for 48 h, followed by detection of cell viability with CCK-8 reagents.

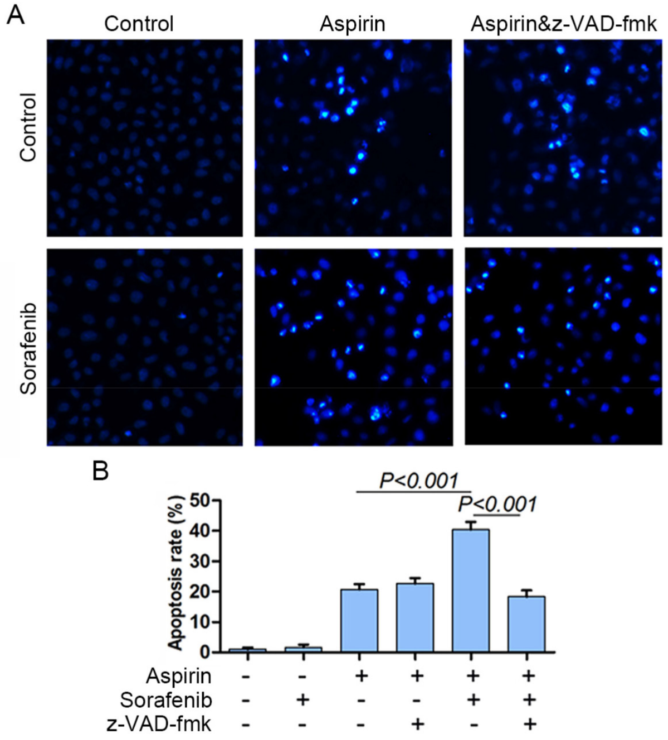

Supplementary Figure S10: Combination of aspirin and sorafenib induces HepG2 cells apoptosis. HepG2 cells were treated with or without 5 mM aspirin, 5 μM sorafenib and the caspase inhibitor z-VAD-fmk (20 μM) for 48 h, followed by detection of apoptosis with Hoechst 33342. The apoptosis rate was plotted.

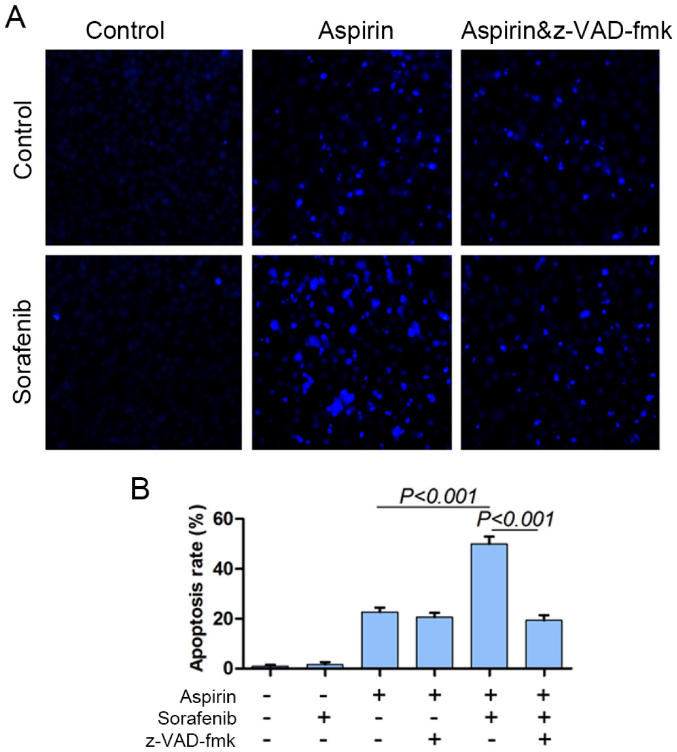

Supplementary Figure S11: Combination of aspirin and sorafenib induces SW480 cells apoptosis. SW480 cells were treated with or without 5 mM aspirin, 5 µM sorafenib and the caspase inhibitor z-VAD-fmk (20 µM) for 48 h, followed by detection of apoptosis with Hoechst 33342. The apoptosis rate was plotted. Western blot analysis of caspase-7 cleavage was also shown.

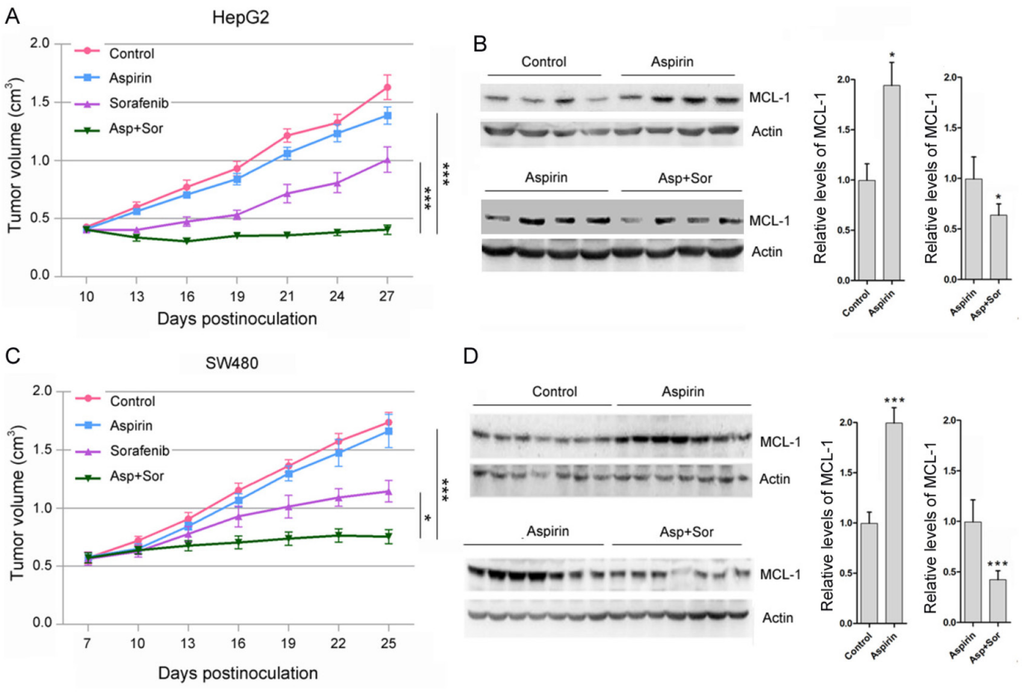

Supplementary Figure S12: Combination of aspirin and sorafenib inhibits tumor growth and MCL-1 expression. 5×10^6 HepG2 (**A**) or SW480 (**C**) cells were injected into Balb/c mice. When tumors became palpable, the mice were randomly assigned into different groups (n = 10 for each group), and treated with aspirin (100 mg/kg/day), sorafenib (15 mg/kg/day), or both daily. Tumor growth was monitored. The levels of MCL-1 in randomly selective tumor samples were also detected by western blotting (**B, D**). *$p < 0.05$. ***$p < 0.01$.
